# Frequency of PCV-2 viremia in nursery piglets from a Spanish swine integration system in 2020 and 2022 considering PRRSV infection status

**DOI:** 10.1186/s40813-024-00354-0

**Published:** 2024-01-16

**Authors:** Mònica Sagrera, Laura Garza-Moreno, Marina Sibila, Salvador Oliver-Ferrando, Sonia Cárceles, Carlos Casanovas, Patricia Prieto, Alberto García-Flores, David Espigares, Joaquim Segalés

**Affiliations:** 1 IRTA. Programa de Sanitat Animal, Centre de Recerca en Sanitat Animal (CReSA, IRTA-UAB), Campus de La UAB, 08193 Bellaterra, Cerdanyola del Vallès, Spain; 2 Unitat Mixta d’Investigació IRTA-UAB en Sanitat Animal, Centre de Recerca en Sanitat Animal (CReSA), Campus de la Universitat Autònoma de Barcelona (UAB), 08193 Bellaterra, Barcelona, Spain; 3 Ceva Salud Animal, Avenida Diagonal, 609-615, 08028 Barcelona, Spain; 4 WOAH Collaborating Center for Research and Control of Emerging and Re-Emerging Pig Diseases (IRTA-CReSA), 08193 Bellaterra, Barcelona, Spain; 5 Inga Food S.A., Ronda de Poniente, 9, 28760 Tres Cantos, Madrid, Spain; 6 Departament de Sanitat i Anatomia Animals, Facultat de Veterinària, UAB, 08193 Bellaterra, Barcelona, Spain

**Keywords:** Porcine circovirus 2 (PCV-2), Porcine reproductive and respiratory syndrome virus (PRRSV), Viremia, Detection frequency, Spain

## Abstract

**Background:**

Porcine circovirus 2 (PCV-2) poses a significant economic threat for the swine industry, causing a range of diseases collectively referred to as porcine circovirus diseases (PCVDs). Despite PCV-2 vaccine effectiveness, the need for monitoring infectious pressure remains. PCV-2 coinfection with other pathogens like porcine reproductive and respiratory syndrome virus (PRRSV) can exacerbate disease severity and lead to PCV-2-systemic disease cases. Monitoring both PRRSV and PCV-2 in co-infected farms is crucial for an effective management and vaccination programs. The present cross-sectional study aimed to determine PCV-2 antibody levels in piglets at weaning and PCV-2 and PRRSV viremia in pooled serum samples at weaning (vaccination age) and at 6 and 9 weeks of age from a Spanish swine integration system in 2020 (48 farms) and in 2022 (28 out of the 48 analysed previously).

**Results:**

The frequency of PCV-2 detection in pools of piglet sera was 2.1% (2020) and 7.1% (2022) at vaccination age but increased at the end of the nursery period (10.4% in 2020 and 39.3% in 2022) in both years. Co-infections between PCV-2 and PRRSV were detected in a significant proportion of PRRSV positive farms (15% in 2020, and 60% in 2022). PCV-2 antibody levels (ELISA S/P ratios) at weaning were lower in PCV-2 qPCR positive farms at different sampling time-points (0.361 in 2020 and 0.378 in 2022) compared to PCV-2 qPCR negative ones (0.587 in 2020 and 0.541 in 2022). The 28 farms tested both years were classified in four different epidemiological scenarios depending on their PCV-2 virological status. Those PCV-2 qPCR negative farms in 2020 that turned to be positive in 2022 had a statistically significant increase of PRRSV RT-qPCR detection and a PCV-2 antibody levels reduction, facts that were not observed in the rest of the scenarios.

**Conclusion:**

This epidemiological study in farms from the same integration system determined the occurrence, in 2020 and in 2022, of PCV-2 and PRRSV infections in piglets during the nursery period by using pooled serum samples.

**Supplementary Information:**

The online version contains supplementary material available at 10.1186/s40813-024-00354-0.

## Background

Porcine circovirus 2 (PCV-2) is considered one of the most economically important pathogens for the swine industry [1]. This virus is ubiquitous in most pig farms, being the causative agent of the so-called porcine circovirus diseases (PCVDs), which include the PCV-2 systemic disease (PCV-2-SD), the PCV-2 reproductive disease (PCV-2-RD), PCV-2 porcine dermatitis and nephropathy syndrome (PDNS) and the PCV-2 subclinical infection (PCV-2-SI) [2, 3].

PCVDs can have a variable impact depending on the PCV-2 immunological and epidemiological herd status, being most of them (except PCV-2-SI) linked to several clinical signs and lesions [4]. Among them, the principal recognized clinical condition is PCV-2-SD, which causes weight loss and wasting, and respiratory and digestive disorders can regularly be observed [3]. The PCV-2-SI, the most common PCVD nowadays, has an economic impact associated with a decrease of 10 to 40 g in average daily weight gain (ADWG) [5, 6]. Therefore, this condition seems to be the most economically impactful for the swine industry [4].

Although PCV-2 vaccines help reducing economic losses attributed to PCVDs, they are not able to completely prevent the viral infection [7–9]. In addition, mass vaccination against PCV-2 has contributed to an overall reduction of herd immunity over time, resulting in batches of animals with no virus exposure from weaning to slaughterhouse [10]. Some pig batches may reach the slaughterhouse being almost seronegative or having a low number of animals seroconverting [4, 10]. Therefore, it is important to monitor infectious pressure, especially at early ages of life, since it may allow re-evaluating control measures applied against PCV-2, such as determining the optimal age for piglet vaccination and the convenience of vaccinating the breeding herd.

Low maternally derived antibody (MDA) titres against PCV-2 and early infections in piglets together with sow viremia around farrowing are known risk factors to increase the likelihood of subsequent development of PCV-2-SD once maternally derived immunity (MDI) is waned [11–14]. Currently, piglet vaccination against PCV-2 is commonly applied around weaning (3–4 weeks of age), since at that age the levels of passive immunity can be overcome without causing significant vaccine intake interference [1, 15].

Although different experimental and field studies have demonstrated the performance of PCV-2 vaccines reducing viremia, clinical signs and/or microscopic lesions in presence of MDA, a proportion of pigs may still develop PCV-2-SI [16–19]. Importantly, the percentage of early infections depends on the balance between the level of MDI and the infectious pressure existing on a particular farm and batch, as well as co-infections with different pathogens [20]. The intensification of the swine industry in the last three decades has favoured more complex clinical presentations due to co-infections, with PCV-2 being found alongside porcine reproductive and respiratory syndrome virus (PRRSV), porcine parvovirus 1, and *Mycoplasma hyopneumoniae*, among others [21].

PRRSV has been considered one of the most important viruses causing disease in pigs and great economic losses worldwide [22–25]. Furthermore, poor cross immunity has been demonstrated between different PRRSV strains, as the virus exhibits high genetic variability and constantly produces new strains with different virulence [26, 27]. Importantly, both PRRSV and PCV-2 target the host’s immune cells by disrupting their function [28, 29] increasing their susceptibility to other pathogens. Such co-infections could affect their growth performance and the incidence and lethality of associated diseases [28, 29]. Indeed, in some studies, PRRSV has been detected in co-infection in PCV-2-SD cases up to 50%, suggesting that co-infection of these viruses is a significant factor contributing to overt disease expression [30–32]. Moreover, it has been demonstrated that PRRSV infection at the time of PCV-2 vaccination jeopardizes the cellular immune response provided by the vaccine [33]. Thus, the surveillance of PRRSV and PCV-2 in farms enables to establish the most effective management and vaccination programs.

Therefore, the objectives of the present study were (1) to determine and compare the frequency of early PCV-2 viremia and antibody levels in piglets of different subclinically infected farms from a Spanish integration system in 2020 and 2022; and (2) to evaluate the frequency of PRRSV infection in those farms.

## Materials and methods

### Study design and sample collection

Forty-eight commercial farrow-to-weaning or farrow-to-nursery farms without PCV-2-SD-like clinical signs were included in the study in 2020, which represented a total of 1860 tested piglets. From these 48 farms, 28 were tested again in 2022 (1140 piglets). The characteristics of each farm regarding herd size, production system and farrowing batches are presented in detail in Additional file 1: Table S1. Farms were located at different areas of Spain, being Aragón and Castilla-León the most represented areas in both years as these regions have the highest pig density in Spain [34].

**Fig. 1:**
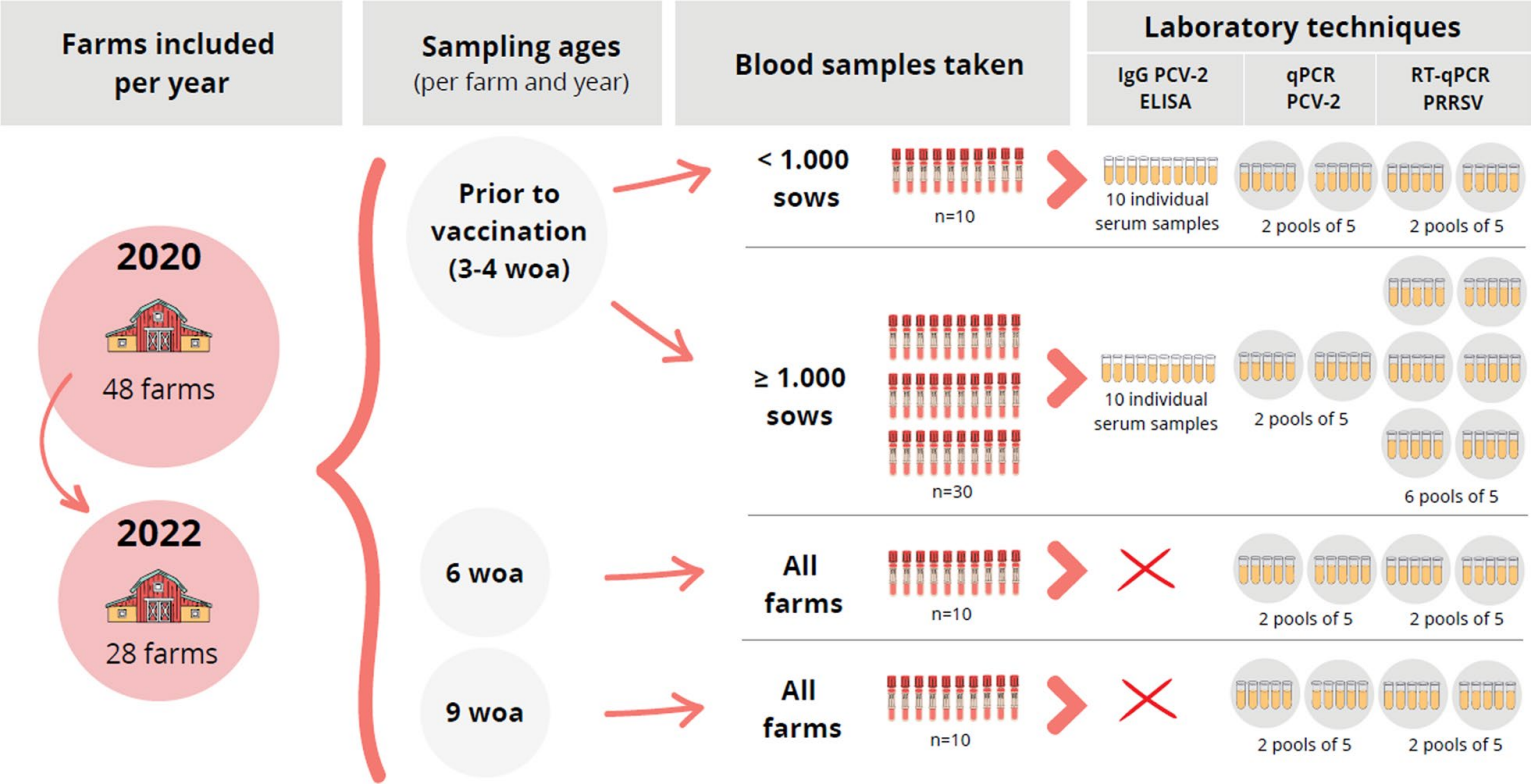
Scheme of the study design, showing the number of farms included in the study in 2020 and in 2022, the different sampled ages, the number of bled pigs depending on the farm size, the laboratory techniques performed (IgG PCV-2 ELISA, qPCR for PCV-2 and RT-qPCR for PRRSV), and the number of samples analysed per each technique. PCV-2: porcine circovirus 2; PRRSV: porcine reproductive and respiratory syndrome virus; woa: weeks of age

In 2020, between January and May, blood samples were collected from apparently healthy piglets at different ages in each selected farm. Sampling methodology included 10 (when farm size was < 1000 sows) or 30 (when farm size was ≥ 1000 sows) blood samples from piglets from different parity sows prior to vaccination (around 3–4 weeks of age), 10 samples at 6 weeks of age (woa), and 10 samples at 9 woa, respectively (Fig. 1). The same sampling procedure was performed in re-tested farms between January and August in 2022. The study followed a cross-sectional design, so, the piglets studied at different time-points were different.

Ten serum samples from the ones taken prior to vaccination per farm were subjected to PCV-2 serology. Such sampling size allowed detecting a theoretical 25% seroprevalence of PCV-2 with 95% confidence (http://www.winepi.net/sp/index.htm). Sera obtained from blood samples taken at all ages were tested by PCV-2 and PRRSV quantitative PCR (qPCR) and RT-qPCR, respectively. Specifically, for PCV-2, 10 sera from all age-groups were tested in two pools of five samples each by qPCR. For PRRSV all available sera were processed through RT-qPCR in pools of five samples. This testing would allow detecting a theoretical frequency of infection of 10% at weaning and 25% at 6 and 9 woa for PRRSV with 95% confidence (http://www.winepi.net/sp/index.htm). These calculations considered the theoretical percentage of detection based on individual samples. Therefore, levels of sensitivity and specificity for detection of these viruses when using pools are expected to be slightly lower compared to individual sample testing [35, 36].

### DNA and RNA extractions and detection of PCV-2 and PRRSV by qPCR methods

Nucleic acids were extracted from 200 μL of each pool (of five samples) using the MagMAX™ Pathogen RNA/DNA Kit (Applied Biosystems) following the manufacturer’s instructions. Negative controls were included to assess potential contamination during extraction.

To detect and quantify the PCV-2 load, a commercial qPCR assay (LSI VetMAX™ Porcine Circovirus Type 2 Quantification, Thermo Fisher Scientific) was used. Each qPCR plate included a negative control and an internal positive control (IPC) to monitor extraction and amplification procedures. Serum pools with < 1.0 × 10^4^ PCV-2 genome copies/mL were considered positive but non-quantifiable. Pools with > 1.0 × 10^4^ PCV-2 genome copies/mL were considered positive and quantifiable. Finally undetermined sample pools (Ct value ≥ 40) were considered negative. Viral load was expressed as the mean PCV-2 genome copies/mL of pooled sera. To calculate the average genome copies per mL of pooled sera, those non-quantifiable positive values were given the cut-off value of 1.0 × 10^4^ PCV-2 genome copies/mL. A farm was considered positive when at least one of the tested pools was positive to PCV-2 at any age.

To detect the PRRSV viremia, a commercial RT-qPCR assay (LSI VetMax™ PRRSV EU/NA 2.0, Thermo Fisher Scientific) was used. Each RT-qPCR plate included negative and IPC to monitor extraction and amplification procedures. Results were expressed as positive (Ct < 40) or negative (Ct ≥ than 40) for PRRSV. A farm was considered positive when at least one tested pool was positive to PRRSV at any tested age.

### Indirect ELISA to detect anti-PCV-2 IgG antibodies

Ten serum samples of piglets at 3–4 woa were individually tested by an indirect commercial ELISA assay (Ingezim Circo IgG 11.PCV.K1® assay, INGENASA) following manufacturer’s instructions. All serum samples per farm were run on the same ELISA plate. The optical density (OD) was measured at 450 nm by the Sunrise™ reader (Tecan). Negative and positive cut-offs were calculated in each ELISA plate and results were expressed as mean S/P ratio (OD of sample/OD of positive control for each ELISA plate) per tested farm.

### Statistical analyses

The normal distribution of the quantitative variables (PCV-2 load and PCV-2 ELISA S/P ratios) was checked by the Shapiro Wilk’s test. PCV-2 load values and PCV-2 ELISA S/P ratios were analysed with the non-parametric Kruskal–Wallis test and Dunn’s multiple comparison test (when comparisons were done between sampling points) or Mann–Whitney test (when comparisons were done between 2020 and 2022). Frequency of detection for both pathogens at different ages and between years were compared using Chi Square or Fisher’s exact test. Statistical analyses and graphics were performed with Graphpad®. The significance level (*p*-value) was set at 0.05, and a trend towards statistical significance was set as 0.1.

## Results

### Forty-eight farms tested in 2020

#### PCV-2 and PRRSV infection

Nine of the 48 (18.8%; CI: 7.7–29.8%) farms tested in 2020 had at least one pool positive by PCV-2 qPCR. From the nine positive farms, one (11.1%; CI: 0.0–31.6%) had a qPCR positive pool prior vaccination, five showed positivity at 6 or at 9 woa (55.6%; CI: 23.1–88.0%), and finally three (33.3%; CI: 2.5–64.1%) had positive pools at both 6 and 9 woa (Table 1 and Additional file 1: Figure S1). In these 9 farms, the PCV-2 load ranged from 10^4^ to 10^8^ copies of PCV-2/mL of pooled sera, being the highest ones detected in those farms where PCV-2 infection was detected in two samplings points.

**Table 1:** PCV-2 qPCR and PRRSV RT-qPCR results obtained prior to vaccination (3–4 woa), and at 6 and 9 woa in the 48 farms sampled in 2020, clustering the farms by their PCV-2 qPCR results

PCV-2 qPCR result	Farms (n, %)		Sampling points	Pools (n, %)	PCV-2 load*	PRRSV RT-qPCR Positive farms (n, %)
Positive	9 (18.8%)	1 (2.1%)	Only at 3–4 woa	1 (0.3%)	8.9 × 10^4^	0 (0.0%)
		3 (6.2%)	Only at 6 woa	5 (1.7%)	2.64 × 10^7^ (3.4 × 10^5^–9.1 × 10^7^)	2 (66.7%)
		2 (4.2%)	Only at 9 woa	3 (1.0%)	1.68 × 10^6^ (1.0 × 10^4^–4.99 × 10^6^)	1 (50.0%)
		3 (6.2%)	At 6 and 9 woa	9 (3.1%)	1.06 × 10^8^ (9.8 × 10^4^–7.0 × 10^8^)	0 (0.0%)
Negative	39 (81.2%)		At 3–4, at 6 and at 9 woa	270 (93.8%)	–	17 (43.6%)
Total	48 (100.0%)		–	288 (100.0%)	6.09 × 10^7^ (1.0 × 10^4^ – 7.0 × 10^8^)	20 (41.7%)

PCV-2: porcine circovirus 2; PRRSV: porcine reproductive and respiratory syndrome virus; woa: weeks of age

*Mean (Min–Max) of PCV-2 load was calculated considering only positive (quantifiable and non-quantifiable) serum pools and are expressed in PCV-2 genome copies/mL

Regarding PRRSV, 20 (41.7%; CI: 27.7–55.6%) of the 48 tested farms had at least one pool positive by RT-qPCR. From these 20 PRRSV positive farms, only 3 (15%; CI: 0.0–30.6%) were also PCV-2 qPCR positive. Co-infection between PRRSV and PCV-2 was detected in pools from sera collected at 6 woa (2 farms) or 9 woa (1 farm).

#### PCV-2 IgG antibody levels in serum prior to PCV-2 vaccination

Globally, farms with pools positive to PCV-2 qPCR (n = 9) showed lower (*p* < 0.1) S/P ratios  (0.529 ± 0.287) than negative ones (0.587 ± 0.286). Specifically, farms with serum pools positive by PCV-2 qPCR at two sampling points (6 and 9) had statistically significant lower PCV-2 IgG ELISA S/P values than those with pools positive only at 6 or 9 woa (*p* < 0.05) (Table 2). However, the coefficient of variation (CV) of these S/P values was high in all groups (Table 2).

**Table 2:** PCV-2 IgG ELISA mean S/P ratios obtained prior to vaccination (3–4 woa), grouped based on PCV-2 qPCR results at 3–4, 6 and 9 woa in the 48 farms sampled in 2020

	PCV-2 qPCR	PCV-2 IgG ELISA prior to vaccination	
Result	Sampling time-point	S/P ratio	
		X̅ ± SD	CV (%)
Positive	Only at 3–4 woa	0.549 ± 0.203 ^ab^	37.0
	Only at 6 woa	0.622 ± 0.316 ^a^	50.2
	Only at 9 woa	0.631 ± 0.303 ^a^	48.7
	At 6 and 9 woa	0.361 ± 0.187 ^b^	51.8
Negative	At 3–4, at 6 and at 9 woa	0.587 ± 0.286 ^a^	48.9
Total		0.576 ± 0.259	47.3%

Different superscript indicates statistically significant differences between groups (*p* < 0.05)

CV: Coefficient of variation; ELISA: Enzyme-Linked ImmunoSorbent Assay; PCV-2: porcine circovirus 2; woa: weeks of age; SD: Standard deviation

### Twenty-eight farms tested in 2022

#### PCV-2 and PRRSV infection

From the 28 farms tested in 2022, 12 (42.9%; CI: 24.5–61.2%) had at least one pool positive by PCV-2 qPCR. Most of these farms (n = 11, 91.7%; CI: 76.0%–100.0% ) had positive pools at 9 woa where the peak of viral load (approx. 10^8^ genome copies of PCV-2/mL of pooled sera) was also detected (Table 3 and Additional file 1: Figure S1).

**Table 3:** PCV-2 qPCR and PRRSV RT-qPCR results obtained prior to vaccination (3–4 woa), and at 6 and 9 woa in the 28 farms sampled in 2022, clustering the farms by their PCV-2 qPCR results

PCV-2 qPCR result	Farms (n, %)		Sampling points	Pools (n, %)	PCV-2 load*	PRRSV RT-qPCR
						Positive farms (n, %)
Positive	12 (42.9%)	1 (3.6%)	Only at 6 woa	2 (1.2%)	6.6 × 10^5^ (1 × 10^4^–1.3 × 10^6^)	1 (3.6%)
		5 (17.9%)	Only at 9 woa	8 (4.8%)	1.1 × 10^8^ (1 × 10^4^ – 8.3 × 10^8^)	4 (14.3%)
		4 (14.3%)	At 6 and 9 woa	12 (7.1%)	1.2 × 10^6^ (1 × 10^4^ – 9.5 × 10^6^)	3 (10.7%)
		1 (3.6%)	At 3–4 and 9 woa	3 (1.8%)	8.3 × 10^7^ (1.7 × 10^4^ – 2.3 × 10^8^)	1 (3.6%)
		1 (3.6%)	At 3–4, at 6 and at 9 woa	4 (2.4%)	3.4 × 10^6^ (1 × 10^4^ –1.3 × 10^7^)	0 (0.0%)
Negative	16 (57.1%)		At 3–4, at 6 and at 9 woa	139 (82.7%)	–	6 (21.4%)
Total	28 (100.0%)		–	168 (100.0%)	4.1 × 10^7^ (1 × 10^4^ – 8.3 × 10^8^)	15 (53.6%)

PCV-2: porcine circovirus 2; PRRSV: porcine reproductive and respiratory syndrome virus; woa: weeks of age; * Mean (Min–Max) of PCV-2 load was calculated considering only positive (quantifiable and non-quantifiable) serum pools and are expressed in PCV-2 genome copies/mL

Fifteen (53.6%; CI: 35.1–72.0%) out of these 28 re-sampled farms had at least one PRRSV RT-qPCR positive pool. From these 15, 9 (n = 60%; CI: 35.2–84.8%) were also positive to PCV-2.

#### PCV-2 IgG antibody levels in serum prior to PCV-2 vaccination

PCV-2 IgG ELISA S/P ratios in farms with qPCR positive serum pools (0.517 ± 0.315) were lower than the ones obtained in the negative ones (0.541 ± 0.285), although not being statistically different. Within the PCV-2 qPCR positive farms, those with pools positive at 3–4 woa had significantly lower S/P ratios than the ones positive only at 9 woa (Table 4). However, a high variability was observed in all groups as CV was higher than 50% in all groups (Table 4).

**Table 4:** PCV-2 IgG ELISA mean S/P ratios obtained prior to vaccination (3–4 woa), grouped based on PCV-2 qPCR results at 3–4, 6 and 9 woa in the 28 farms sampled in 2022

	PCV-2 qPCR	PCV-2 IgG ELISA prior to vaccination	
Result	Sampling time-point	S/P ratio	
		X̅ ± SD	CV (%)
Positive	Only at 6 woa	0.432 ± 0.298 ^ab^	69.0
	Only at 9 woa	0.647 ± 0.345 ^a^	53.4
	At 3–4 and 9 woa	0.280 ± 0.205 ^b^	73.3
	At 6 and 9 woa	0.470 ± 0.260 ^ab^	55.4
	At 3–4, at 6 and at 9 woa	0.378 ± 0.204 ^ab^	54.0
Negative	At 3–4, at 6 and at 9 woa	0.541 ± 0.285 ^ab^	52.7
Total	0.530 ± 0.298	56.1%	

Different superscript indicates statistically significant differences between groups (p < 0.05)

CV: Coefficient of variation; ELISA: Enzyme-Linked ImmunoSorbent Assay; PCV-2: porcine circovirus 2; woa: weeks of age; SD: Standard deviation

#### PCV-2 and PRRSV infection and PCV-2 IgG antibodies in 28 farms in 2020 and 2022: definition of epidemiological scenarios

The 28 farms tested both years were classified considering the PCV-2 virological results into four different epidemiological scenarios (Additional file 1: Figure S1).

POS20-POS22: farms PCV-2 qPCR positive in both years (n = 4, 14.3%). Although the 4 farms were positive in both years, two of these farms in 2022 had a lower frequency of detection and the PCV-2 load was non-quantifiable (Additional file 1: Table S2). Regarding PRRSV viremia, 3 out of these 4 farms maintained the status in both years and the remaining one (SP-19) changed from RT-qPCR negative to positive.

POS20-NEG22: farms PCV-2 qPCR positive in 2020 but negative in 2022 (n = 1, 3.6%). This scenario was composed only by one farm that in 2020 had pools positive to PCV-2 prior to vaccination but in 2022 all the tested pools were negative. This farm was RT-qPCR negative for PRRSV in both years.

NEG20-POS22: farms PCV-2 qPCR negative in 2020 that turned to be positive in 2022 (n = 8, 28.6%). In this scenario, a statistically significant increase of PCV-2 detection frequency (*p* < 0.05) was detected from 2020 to 2022, mainly among the 6 and 9 woa groups (Additional file 1: Table S3). Within this scenario, the number of PRRSV RT-qPCR positive farms was significantly higher (*p* < 0.05) in 2022 (7 out of 8, 87.5%; CI: 64.6%–100.0%) compared to 2020 (3 out of 8, 37.5%; 3.95%–71.05%).

NEG20-NEG22: farms that were negative to PCV-2 qPCR both years (n = 15, 53.6%). Nine of these farms were PRRSV RT-qPCR positive in 2020 and five of them tested positive again in 2022. An additional farm turned positive that year (Additional file 1: Table S4).

Regarding PCV-2 IgG detection, the mean ELISA S/P ratios increased from 2020 to 2022 in scenarios POS20-POS22 and POS20-NEG22, while these decreased over the same period for scenarios NEG20-POS22 and NEG20-NEG22 (Table 5). These variations were only significant for NEG20-POS22 farms. When comparing scenarios within each year, only mean S/P ratios were significantly lower (p < 0.05) in POS20-POS22 farms compared to NEG20-POS22 and NEG20-NEG22 herds in 2020.

**Table 5:** PCV-2 IgG ELISA mean S/P ratios obtained prior to vaccination (3–4 woa) in the 28 farms sampled in 2020 and 2022, grouping the farms in the four stablished scenarios considering PCV-2 detection in both studied years

Defined scenarios	PCV-2 IgG ELISA S/P ratio before vaccination (3–4 woa)			
	2020		2022	
	X̅ ± SD	CV (%)	X̅ ± SD	CV (%)
POS20-POS22	0.397 ± 0.197 ^a^ ^A^	49.1	0.599 ± 0.372 ^a^ ^A^	62.0
POS20-NEG22	0.549 ± 0.203 ^a^ ^AB^	37.0	0.686 ± 0.263 ^a^ ^A^	38.4
NEG20-POS22	0.628 ± 0.286 ^a^ ^B^	45.5	0.476 ± 0.275 ^b^ ^A^	57.9
NEG20-NEG22	0.639 ± 0.342 ^a^ ^B^	53.4	0.531 ± 0.284 ^a^ ^A^	53.6
Total	0.598 ± 0.315	56.6	0.530 ± 0.298	56.1

Different superscript lowercase letters indicate statistically significant differences between years for each scenario, and the uppercase ones indicate statistically significant differences between scenarios in each year (p < 0.05)s

CV: Coefficient of variation; ELISA: Enzyme-Linked ImmunoSorbent Assay; PCV-2: porcine circovirus 2; woa: weeks of age; SD: Standard Deviation

## Discussion

The economic impact of PCVDs has been considerably reduced since the advent of PCV-2 vaccines [6, 7, 9]. Such control of overt diseases associated with PCV-2 lead to the fact that the most frequent presentation nowadays is PCV-2-SI [3, 9, 37], which implies the interest to monitor the infection despite the lack of clinical signs. Therefore, the present study sought to determine the PCV-2 and PRRSV frequency of detection in nursery pigs from 48 commercial farms in Spain in 2020 with no clinical signs associated to PCVDs and to evaluate the epidemiological situation of these two pathogens in a proportion of these farms (n = 28) two years later.

In both years, there were farms with PCV-2 qPCR positive results in 3-to-9-week-old pigs with high PCV-2 loads, up to 1.0 × 10^8^ copies/mL of pooled sera, which might potentially fit with a tentative diagnosis of PCV-2-SD when following the different thresholds proposed [3, 12, 38]. However, these thresholds were established with different qPCR methods compared to those used nowadays [4],therefore, it is very likely that obtained values (from pools of five sera) could be indicative of subclinical infections considering the lack of overt clinical signs in the herd at the moment of sampling. It is important to remark that a final diagnosis of PCV-2-SD must be established by means of histopathological lymphoid lesions and detection of PCV-2 within these lesions [4],therefore, the unequivocal diagnosis of PCV-2-SD could not be established based only on qPCR results.

In the 48 farms analysed in 2020, the frequency of PCV-2 detection in pools of piglet sera was very low at vaccination age (3–4 woa, 2.1% of the farms, n = 1/48) but increased up to 10.4% at the end of the nursery period (n = 5/48). The obtained low prevalence in suckling pigs agrees with previous epidemiological studies performed in Europe that described PCV-2 early viremia in piglets from endemically infected farms as fairly uncommon [39, 40]. Across the 28 farms analysed in 2022, the frequency of PCV-2 detection was higher towards the end of the nursery period (n = 11/28, 39.3% of the farms), despite being lower at vaccination age (n = 2/28, 7.1% of the farms). Results from both years are relatively low compared with the ones obtained in other studies performed in North-America [41, 42]. However, these latter studies were published at a time when PCV-2 vaccination was not as extensive as nowadays. Additionally, all these studies tested individual samples, whereas the present study is based on pooled samples to mimic usual field sampling and monitoring conditions used by swine veterinarians in different parts of the world. Studies in pools likely imply a reduction in the observed PCV-2 detection frequency [35, 36], but it was adopted to have the possibility to screen the higher number of farms/animals possible based on the epidemiological criteria set (theoretical frequency of infection of 25% for PCV-2 at all tested ages, and 10% at weaning and 25% at 6 and 9 woa for PRRSV with 95% confidence). In turn, this represents one of the limitations of this study, since we would not be able to detect epidemiological situations in which lower percentages of infection with these pathogens may occur.

Maternally derived immunity transferred to piglets was evaluated in terms of antibody levels at vaccination age (3–4 weeks of age) both years, and antibody S/P ratios were moderate-to-low and highly variable (close to 50% CV). Specifically, in both years, the ELISA S/P ratio tended to be lower when PCV-2 viremia was detected at more than one sampling time-point in a farm, than when viremia was not detected or was detected only in one sampling time-point. This could reinforce the statement that MDI has a protective effect against PCV-2 infection, and the moderate-to-low values observed fit with the described epidemiological change of PCV-2 infection due to vaccination pressure [4, 43]. These results are also in line with a previous study indicating that protection conferred by MDA is titre dependent, so its presence does not guarantee full protection against the infection, as previously described [44–46].

Additionally, the number of farms positive to PRRSV increased from almost 42% in 2020 up to 54% in 2022. In January 2020, a PRRSV-1 strain of increased virulence (commonly known as Rosalia), which was characterised by high abortion rates and increase mortality rates in weaners, was reported in North-Eastern (NE) Spain [27, 47]. The NE and its surrounding regions concentrate almost half of the Spanish pig farms and corresponds to the region from most of the farms tested in the present study [34]. This situation would probably explain the increase of farms positive to PRRSV, despite clinical signs due to this viral infection were not seen at the time when samplings were performed.

As previously mentioned, PRRSV and PCV-2 target the host’s immune cells by disrupting their function, and they have been detected co-existing in some PCV-2-SD cases, emphasizing that such co-infection can be a main driver for overt PCVD expression [29, 32]. Therefore, it is not surprising to have cases of PCV-2 and PRRSV co-infection in the nursery phase (in 15.0% and 60.0% of PRRSV positive farms in 2020 and 2022, respectively), as it has been already described [32], despite not having clinical problems in analysed farms.

To further examine the PRRSV and PCV-2 farm status co-evolution in more detail, the results from the 28 farms that were tested in 2020 and in 2022 were compared, classifying them into four epidemiological scenarios. In PCV-2 POS20-POS22 and NEG20-NEG22 ones, the total PRRSV detection frequency decreased (from 13.9 to 8.3%, and from 29.7 to 24.6%, respectively). The same happened with the PCV-2 detection frequency (from 45.8 to 33.3%) and the PCV-2 load (from the 8.76 × 10^7^ to 1.13 × 10^7^ copies of PCV-2 DNA/mL of pooled sera) in the PCV-2 POS20-POS22 scenario. Meanwhile, in the NEG20-POS22 scenario, PRRSV and PCV-2 detection frequency increased (from 22.1% to 50.0%, and from negative to 43.8%, respectively). In the POS20-POS22 scenario, the overall increase in the average ELISA S/P ratios, together with the reduction in PCV-2 load and PCV-2 and PRRSV detection frequencies between years, could reinforce the previously mentioned suggestion of the protective effect of MDI. However, in the NEG20-POS22 scenario, lower levels of anti-PCV-2 IgG antibodies from 2020 to 2022 could have been facilitated by the PRRSV infection in the farms, since it has a suppressive effect of the innate immunity and might jeopardize the pig’s immune response [29, 32, 48–50]. PRRSV influences the activation of the specific immune response, and an early PRRSV infection could compromise the efficacy of PCV-2 vaccines due to its detrimental effect on the development of naïve T cells, while it could negatively influence on the immune response to other pathogens [32, 33].

A similar reduction in the ELISA S/P ratios between years was observed in NEG20-NEG22, which could be due to an overall reduction in herd immunity due to the high efficacy of PCV-2 vaccines decreasing infection pressure in the farms. In such scenario, sow vaccination could be a good option to avoid the putative future occurrence of PCV-2-SD in piglets [4, 6].

## Conclusion

The present epidemiological study describes the PCV-2 S/P ratios at weaning and the PCV-2 and PRRSV frequency of detection at 3–4, 6 and 9 woa in piglets from commercial swine farms from an integration system in two different periods (2020 and 2022). The results obtained revealed a higher frequency of PRRSV and PCV-2 detection in 2022 compared to 2020, including a higher incidence of co-infections. This evolution coincided with the appearance of highly virulent strains of PRRSV in Spain. We identified four epidemiological scenarios related to these infections, emphasizing the importance of continuous monitoring and adaptive measures for effective PCV-2 vaccination practices, particularly in light of early PCV-2 and PRRSV co-infections.

### Supplementary Information

**Additional file 1**. Supplementary materials of the study "Frequency of PCV-2 viremia in nursery piglets from a Spanish swine integration system in 2020 and 2022 considering PRRSV infection status". 

## Data Availability

All data generated or analysed during this study are included in this published article [and its Additional file 1.].

## Abbreviations

ELISA: Enzyme-linked immunosorbent assay
PCV-2: Porcine circovirus 2
PRRSV: Porcine reproductive and respiratory virus
qPCR: Quantitative polimerase chain reaction
RT-qPCR: Retrotranscriptase quantitative polimerase chain reaction
woa: Weeks of age
