# Supplementary material for: Frequency of PCV-2 viremia in nursery piglets from a Spanish swine integration system in 2020 and 2022 considering PRRSV infection status
Source: Porcine Health Manag. 2024 Jan 16;10:4. doi: 10.1186/s40813-024-00354-0 (PMC10792786; doi:10.1186/s40813-024-00354-0)
Supplement: Supplementary file 1 — Additional file 1. Supplementary materials of the study "Frequency of PCV-2 viremia in nursery piglets from a Spanish swine integration system in 2020 and 2022 considering PRRSV infection status". [file 40813_2024_354_MOESM1_ESM.docx]

***Supplementary Table 1.*** Characteristics of farms included in this study based on herd size, type of production system, farrowing batches, and piglet PCV-2 vaccination age in 2020. Those farms re-sampled two years later are also noted.

| **Farm information** | | | | | **Sampling repeated in 2022** |
| --- | --- | --- | --- | --- | --- |
| **Farm ID** | **Herd size** | **Production system** | **Farrowing batch** | **Age at PCV-2 vaccination**  **(woa)** |  |
| SP-1 | 2500 | 1 site | weekly | 4 woa | Yes |
| SP-2 | 1000 |  |  | 4 woa | No |
| SP-3 | 2000 |  |  | 4 woa | Yes |
| SP-4 | 500 |  |  | 3 woa | Yes |
| SP-5 | 2500 |  |  | 4 woa | Yes |
| SP-6 | 1000 |  |  | 4 woa | Yes |
| SP-7 | 500 |  |  | 4 woa | No |
| SP-8 | 500 |  |  | 4 woa | No |
| SP-9 | 750 |  |  | 4 woa | No |
| SP-10 | 750 |  |  | 4 woa | Yes |
| SP-11 | 1100 |  |  | 3 woa | Yes |
| SP-12 | 800 |  |  | 4 woa | Yes |
| SP-13 | 3400 |  | 3-weeks | 4 woa | Yes |
| SP-14 | 1650 |  |  | 3 woa | Yes |
| SP-15 | 300 |  |  | 4 woa | No |
| SP-16 | 550 |  | 5-weeks | 4 woa | No |
| SP-17 | 1400 |  |  | 3 woa | No |
| SP-18 | 800 |  |  | 3 woa | No |
| SP-19 | 800 | 2 sites ^*^ | weekly | 4 woa | Yes |
| SP-20 | 700 |  |  | 3 woa | No |
| SP-21 | 230 |  |  | 3 woa | No |
| SP-22 | 200 |  |  | 4 woa | No |
| SP-23 | 1000 |  |  | 3 woa | Yes |
| SP-24 | 800 |  |  | 4 woa | No |
| SP-25 | 900 |  |  | 3 woa | No |
| SP-26 | 900 |  |  | 4 woa | Yes |
| SP-27 | 900 |  |  | 4 woa | No |
| SP-28 | 1400 |  |  | 3 woa | Yes |
| SP-29 | 700 |  |  | 3 woa | Yes |
| SP-30 | 1663 |  |  | 4 woa | Yes |
| SP-31 | 1196 |  |  | 3 woa | Yes |
| SP-32 | 1100 |  |  | 4 woa | No |
| SP-33 | 700 |  |  | 3 woa | Yes |
| SP-34 | 531 |  |  | 3 woa | Yes |
| SP-35 | 1700 |  |  | 4 woa | No |
| SP-36 | 1000 |  |  | 4 woa | Yes |
| SP-37 | 1233 |  | 2-weeks | 4 woa | Yes |
| SP-38 | 572 |  | 3-weeks | 4 woa | Yes |
| SP-39 | 525 |  |  | 4 woa | Yes |
| SP-40 | 634 |  |  | 4 woa | Yes |
| SP-41 | 361 |  |  | 4 woa | No |
| SP-42 | 2134 |  |  | 3 woa | Yes |
| SP-43 | 1350 |  |  | 3 woa | No |
| SP-44 | 1000 |  |  | 3-4 woa | Yes |
| SP-45 | 500 |  |  | 3 woa | Yes |
| SP-46 | 1700 |  |  | 3-4 woa | No |
| SP-47 | 680 |  | 5-weeks | 3-4 woa | Yes |
| SP-48 | 600 |  |  | 3 woa | No |

^*^ 2 sites farm were considered those having sites I (gestation and farrowing) and II (nursery) in the same farm; woa: weeks of age; PCV-2: porcine circovirus 2.

***Supplementary Table 2.*** PCV-2 and PRRSV qPCR detection frequency, and PCV-2 load, at different time-points in 2020 and 2022 of the farms grouped in scenario POS20-POS22.

| **Age (woa)** | **Year** | **PCV-2 qPCR** | | | | **PRRSV qPCR** | | |
| --- | --- | --- | --- | --- | --- | --- | --- | --- |
|  |  | **Positive farms (n)** | **Positive pools (n)** | **Viral load**  PCV-2 copies/mL of serum  X̅ (Min -Max) | **Detection frequency** | **Positive farms**  **(n)** | **Positive pools (n)** | **Detection frequency** |
| **3-4 woa** | **2020** | 0/4 | 0/8 | - | 0% **^a^** | 1/4 | 1/20 | 5.0%  (CI: 0.0% - 14.6%) |
|  | **2022** | 0/4 | 0/8 | - | 0% **^a^** | 1/4 | 1/20 | 5.00%  (CI: 0.0% - 14.6%) |
| **6 woa** | **2020** | 3/4 | 3/8 | 7.67x10^6^  (1.60x10^6^ - 1.60x10^7^) | 37.5% **^a^**  (CI: 4.0% - 71.1%) | 1/4 | 2/8 | 25.0%  (CI: 0.0% - 55.0%) |
|  | **2022** | 2/4 | 2/8 | 1.05 x10^4^  (<1x10^4^ – 1.10x10^4^) | 25.0% **^a^**  (CI: 0.0% - 55.0%) | 0/4 | 0/8 | 0% |
| **9 woa** | **2020** | 4/4 | 8/8 | 1.18x10^8^  (<1x10^4^ – 7.00x10^8^) | 100% **^b^** | 1/4 | 2/8 | 25.0%  (CI: 0.0% - 55.0%) |
|  | **2022** | 4/4 | 6/8 | 1.51x10^7^  (<1x10^4^ – 8.10x10^7^) | 75.0% **^b^**  (CI: 45.0% - 100.0%) | 1/4 | 2/8 | 25.0%  (CI: 0.0% - 55.0%) |
| **Total 2020** | | 4/4 | 11/24 | 8.76x10^7^ *  (<1x10^4^ – 7.00x10^8^) | 45.8%  (CI: 25.9% - 65.8%) | 1/4 | 5/36 | 13.9%  (CI: 2.6% - 25.2%) |
| **Total 2022** | | 4/4 | 8/24 | 1.13x10^7^ **  (<1x10^4^ – 1.13x10^7^) | 33.3%  (CI: 14.5% - 52.2%) | 2/4 | 3/36 | 8.3%  (CI: 0.0% - 17.4%) |

PCV-2: porcine circovirus type two; PRRSV: porcine reproductive and respiratory virus; woa: weeks of age; CI: Interval of Confidence. Ranges (Min-Max) have been calculated considering only positive pools of samples and those are expressed in PCV-2 genome copies/mL. Different superscript letters indicate statistically significant differences in PCV-2 prevalence within time-points and ages (p<0.05), and a statistically significant trend is marked with */** (p<0.1).

***Supplementary Table 3.*** PCV-2 and PRRSV qPCR detection frequency, and PCV-2 load, at different time-points in 2020 and 2022 of the farms grouped in scenario NEG20-POS22.

| **Age (woa)** | **Year** | **PCV-2 qPCR** | | | | **PRRSV qPCR** | | |
| --- | --- | --- | --- | --- | --- | --- | --- | --- |
|  |  | **Positive farms (n)** | **Positive pools (n)** | **Viral load**  PCV-2 copies/mL of serum  X̅ (Min -Max) | **Detection frequency** | **Positive farms (n)** | **Positive pools (n)** | **Detection frequency** |
| **3-4 woa** | **2020** | 0/8 | 0/16 | - | 0% **^a^** | 3/8 | 7/36 | 19.4% **^A^**  (CI: 6.5% - 32.4%) |
|  | **2022** | 2/8 | 2/16 | 1.35x10^4^  (<1.0x10^4^ – 1.70x10^4^) | 12.5% **^a^**  (CI: 0.0% - 28.7%) | 3/8 | 12/36 | 33.3% **^A^**  (CI: 17.9 % - 48.7%) |
| **6 woa** | **2020** | 0/8 | 0/16 | - | 0 % **^a^** | 2/8 | 4/16 | 25.00% **^A*^**  (CI: 3.8% - 46.2%) |
|  | **2022** | 3/8 | 7/16 | 6.21x10^5^  (<1.0x10^4^ – 1.90x10^6^) | 43.8% **^b^**  (CI: 19.4% - 68.1%) | 5/8 | 10/16 | 62.50% **^A**^**  (CI: 38.8% - 86.2%) |
| **9 woa** | **2020** | 0/8 | 0/16 | - | 0% **^a^** | 2/8 | 4/16 | 25.0% **^A^**  (CI: 3.8% - 46.2%) |
|  | **2022** | 7/8 | 12/16 | 9.14x10^7^  (<1.0x10^4^ – 8.30x10^8^) | 75.0% **^b^**  (CI: 53.8% - 96.2%) | 6/8 | 12/16 | 75.0% **^B^**  (CI: 53.8% - 96.2%) |
| **Total 2020** | | 0/8 | 0/48 | - | 0% **^a^** | 3/8 | 15/68 | 22.1% **^A^**  (CI: 12.2% - 31.9%) |
| **Total 2022** | | 8/8 | 21/48 | 5.24x10^7^  (<1.0x10^4^ – 8.30x10^8^) | 43.8% **^b^**  (CI: 29.7% - 57.8%) | 7/8 | 34/68 | 50.0% **^B^**  (CI: 38.1% - 61.9%) |

PCV-2: porcine circovirus type two; PRRSV: porcine reproductive and respiratory virus; woa: weeks of age; CI: Interval of Confidence. Ranges (Min-Max) have been calculated considering only positive pools of samples and those are expressed in PCV-2 genome copies/mL. Different superscript lowercase letters within 2020 and 2022 and different ages rows indicate PCV-2 detection frequency statistically significant differences (p<0.05). The uppercase ones indicate the PRRSV detection frequency statistically significant differences (p<0.05), and a statistically significant trend is marked with */** (p<0.1).

***Supplementary Table 4.*** PCV-2 and PRRSV qPCR detection frequency, and PCV-2 load, at different time-points in 2020 and 2022 of farms grouped in scenario NEG20-NEG22.

| **Age (woa)** | **Year** | **PCV-2 qPCR** | | | | **PRRSV qPCR** | | |
| --- | --- | --- | --- | --- | --- | --- | --- | --- |
|  |  | **Positive farms (n)** | **Positive pools (n)** | **Viral load**  PCV-2 copies/mL of serum  X̅ (Min -Max) | **Detection frequency** | **Positive farms (n)** | **Positive pools (n)** | **Detection frequency** |
| **3-4 woa** | **2020** | 0/15 | 0/30 | - | 0% | 6/15 | 13/58 | 22.4% ^a^  (CI: 11.7% - 33.2%) |
|  | **2022** | 0/15 | 0/30 | - | 0% | 4/15 | 10/58 | 17.2% ^a^  (CI: 7.5% - 27.0%) |
| **6 woa** | **2020** | 0/15 | 0/30 | - | 0% | 5/15 | 9/30 | 30.0% ^ab^  (CI: 13.6% - 46.4%) |
|  | **2022** | 0/15 | 0/30 | - | 0% | 5/15 | 8/30 | 26.7% ^ab^  (CI: 10.8% - 42.5%) |
| **9 woa** | **2020** | 0/15 | 0/30 | - | 0% | 7/15 | 13/30 | 43.3% ^b^  (CI: 25.6% - 61.1%) |
|  | **2022** | 0/15 | 0/30 | - | 0% | 6/15 | 11/30 | 36.7% ^b^  (CI: 19.4% - 53.9%) |
| **Total 2020** | | 0/15 | 0/90 | - | 0% | 9/15 | 35/118 | 29.7%  (CI: 21.4% - 37.9%) |
| **Total 2022** | | 0/15 | 0/90 | - | 0% | 6/15 | 29/118 | 24.6%  (CI: 16.8% - 32.3%) |

PCV-2: porcine circovirus 2; PRRSV: porcine reproductive and respiratory virus; woa: weeks of age; CI: Interval of Confidence. Different superscript letters within 2020 and 2022 and different ages rows indicate PRRS frequency of detection statistically significant tendencies (p<0.1).

***Supplementary Figure 1*.** PCV-2 and PRRSV qPCR results, and PCV-2 IgG ELISA S/P ratios of the 48 farms analysed in 2020 and the 28 analysed again in 2022, ordered by the proposed scenarios.


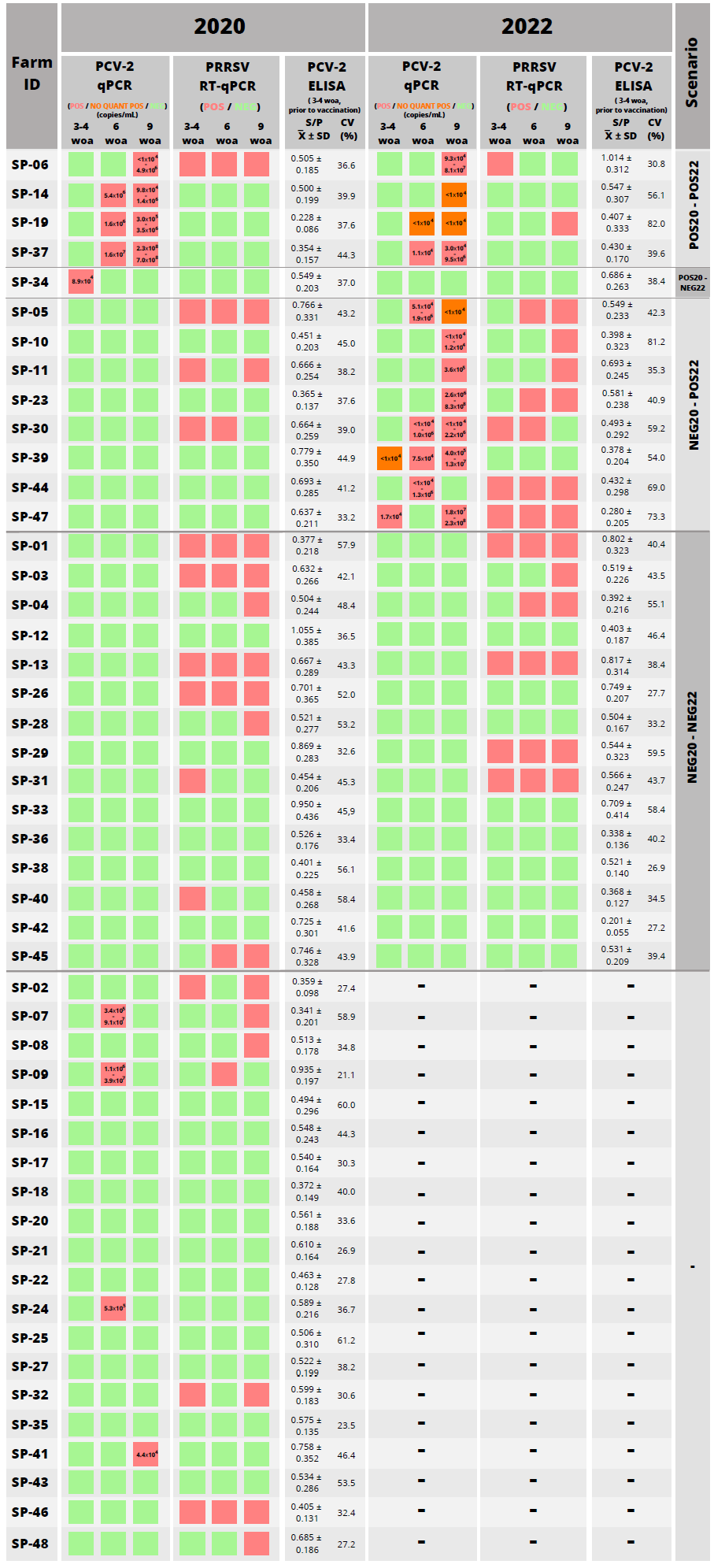


PCV-2: porcine circovirus 2; PRRSV: porcine reproductive and respiratory syndrome virus; woa: weeks of age; POS: positive; NO QUANT POS; non-quantifiable positive; NEG: negative; ELISA: Enzyme-Linked ImmunoSorbent Assay; SD: Standard deviation; CV: Coefficient of variation.
